# Preventable Social Ills

**Published:** 1913-01-18

**Authors:** 


### II. PREVENTABLE SOCIAL ILLS.

### Prevention as a New Art.

The science of community life, whereby men from the highest to the lowest may attain to live together in mutual harmony and well-being, is of comparatively recent origin, but it has already in this country produced very definite results. There are three stages in every movement for the amelioration of social evils. The first is the battle for recognition of facts, and so prone are men to ignore the existence of all that is disagreeable to them, so indignant are they at the unveiling of painful truths, that those who engage in this part of the struggle have by far the hardest part to fill. The first half of the Victorian era witnessed a mighty revolution in thought in regard to the condition of the working classes, that is to say, of nine-tenths of the population. That work, done in the teeth of most bitter opposition from the few thousands who were not horribly discomforted by the abuses laid bare, has been done once and for all. It is now a mere truism that social adjustments and alterations are, not merely desirable in the interests of the many, but absolutely indispensable in the interests of all. Accordingly the last part of the nineteenth century witnessed the progress of the second stage in an organised attack upon disease and wretchedness. Myriad philanthropic agencies were set in motion to this end, and although they proved for the most part inadequate for the required purpose, and some of them even directly contributed to the creation of fresh difficulties, the knowledge gained in this experimental stage is priceless and perhaps attainable in no other way.

It would seem that in the new Georgian era we are beginning to emerge into the third stage of social consciousness, that in which the energies are directed towards the prevention rather than the cure of those physical, moral, and mental diseases under which the nation groans. The simple, wise words of King Edward, which have told for much in the effort to root out tuberculosis, "If preventable, why not prevented?" stand as the motto of the new sociology. The enthusiasm which drew all the wretched sufferers of the country to be treated in hospitals is still stirring, but it begins to concern itself increasingly with the causes which entail this mass of suffering, this strange variety of diseases on normal beings. A kind of national soul is beginning to stir in the land, a soul which will not rest content with a humane rôle towards paupers, but begins to revolt against the existence of pauperism. The world is no longer content with building plague-houses and allowing devoted nuns to sacrifice their lives in heroic deeds of mercy for dying men. The plague-houses are fallen into ruins, and regions of the globe once deemed uninhabitable by reason of yellow fever or malaria have risen into the rank of health resorts. May we not one day watch with equal satisfaction the decay of many a philanthropical scheme designed for the curing of ills at length successfully prevented?

It is a striking and singular testimony to the splendid work already accomplished that poor and rich alike, those who suffer directly and those who suffer but indirectly from the faulty adjustments of the social machine, are in this country moving in the same direction towards similar ends, though it may be with differing notions of what are the best methods of procedure. What is generally known as "catastrophic socialism" has in this country hardly gained admittance and has no root.

True sociology is the science of co-ordinating means to an end in the life of the community for the avoidance of mutual injuries and the promotion of individual freedom. Bearing this definition in mind, it would appear that the Englishman's genius for managing his own affairs has impressed a special character upon the sociology of this country, as compared with the doctrines which dominate other nationalities in their search after social harmony. It is the peculiar distinction of English legislative movements for the betterment of those who lie below the line of well-being that they have, with one or more striking exceptions, sprung from the heart of the people themselves, instead of being dictated by statesmen. All the great movements for the education of youth, for the relief of disease, insanity, and infectious complaints, for the promotion of thrift, for the prevention of drunkenness, for housing the poor, and for a hundred other social improvements, have gone through three stages in this country. First, the theorists have demonstrated the existence of the want or evil to be overcome. Next, voluntary zeal has attempted the remedy, kindling in one locality from the torch set alight in another until a demonstration has been afforded to the country of successful operations too far-reaching to remain under the control of private persons. Lastly, the philanthropic undertaking, no machine-made article, but the product of genuine growth under widely differing conditions, has passed under the control of the State, well adapted to take on yet another form of life under wider opportunities. Had the treatment of the destitute been suffered to proceed along these normal and healthy lines it may be safely prophesied that the blunders committed under the Poor Act of Elizabeth would not have stained our national history; but, unhappily, the voluntary agencies for relieving distress were swept abruptly away with the destruction of the monasteries, and an entirely new and hostile attitude towards the poor was subsequently brought about by men ignorant of the conditions with which they were tampering.

There is no factor in English national life more vital and necessary than the element of personal service which has always attended social progress. The futility of legislation alone to produce amelioration of the race has been demonstrated continuously in the history of every country. It has been illustrated conspicuously, to give but one example, in the history of midwifery. At the time of the agitation in favour of midwifery legislation ten years ago it was pointed out that in no country were midwives more incompetent and ill-instructed than in those where legislation had been earliest obtained for their regulation. It has been evident from the first that the success which has attended the English Midwives Act is due to the admirable training schools provided by voluntary effort. Without the long series of struggles, failures, researches, and triumphs witnessed in our voluntarily supported lying-in hospitals since their foundation 150 years ago the Midwives Act could merely have given a prestige to ignorant blunderers. Even when the third stage is reached and wise legislation has crowned the work long in preparation personal service cannot safely be dismissed. The final stages of reform, which are not final but merely a new beginning, require an even greater watchfulness and zeal than was needed during the experimental phases, and it must be the determination of all who have the interests of their fellows at heart to retain for the people of this country that capacity for minding their own affairs and helping each other which has preserved them from the dread shadow of bureaucracy in their dealings with all sufferers except such as have been relegated to the Poor Law.

The social philosophy which is destined to influence mankind must be based on faith. Unless men believe that good is the great reality, they must for ever rest content with a reign of injustice and with a social outlook which may be defined as "the greater misery of the greater number." Unless men believe in each other they cannot co-operate for their mutual benefit; they cannot work towards a higher future for mankind or watch with sympathy the efforts of their fellows to ascend. Faith is the only lever through which, as the greatest of social reformers once testified, the mountain of so-called "necessary evils" can be removed and cast into the sea.

